# Plant developmental stage drives the assembly and functional adaptability of endophytic microbial communities

**DOI:** 10.3389/fmicb.2025.1492141

**Published:** 2025-05-29

**Authors:** Min Yang, Jindan Wang, Ying Qi, Penghua Gao, Lifang Li, Jianwei Guo, Yongteng Zhao, Jiani Liu, Zebin Chen, Jianrong Zhao, Lei Yu

**Affiliations:** College of Agronomy, Yunnan Key Laboratory of Konjac Biology, Yunnan Urban Agricultural Engineering and Technological Research Center, Kunming University, Kunming, China

**Keywords:** *Amorphophallus muelleri*, seed, plant developmental stage, endophytic microbial communities, function

## Abstract

**Introduction:**

The seeds of *Amorphophallus muelleri* represent a unique category of herbaceous seeds that arise from triploid apomixis. They necessitate an exceptionally protracted maturation phase of 8 months, followed by a dormancy period of 4 months, before they can germinate and give rise to fully formed new plants. Currently, the connection between endophytic microbial communities in *A. muelleri* seeds and the host plant’s development is largely unexplored.

**Methods:**

Herein, we analyzed the temporal dynamics of the endophytic bacterial and fungal communities from seed germination to seedling establishment (seven stages) through amplicon sequencing.

**Results and discussion:**

The results showed that plant developmental stage explained the large variation in endophytic bacterial and fungal communities in *A. muelleri* and that multiple microbial attributes (e.g., α, β-diversity, community composition, and bacterial and fungal ecological networks) are driven by the developmental state of *A. muelleri*. Metagenomic analyses further indicated that the four stages after rooting have higher microbial functional diversity. Microbial functional genes involved in cell wall/membrane/envelope biogenesis, inorganic ion transport and metabolism, and carbon degradation were abundant in *A. muelleri* seeds from Stage 1 to Stage 3 (before rooting). From Stage 4 to Stage 7 (after rooting), microbial functional genes involved in the carbon, nitrogen and phosphorus cycles, starch and sucrose metabolism, and energy production and conversion were more abundant. Coincidentally, more abundant Proteobacteria, and Basidiomycota taxa related to carbon degradation were found in stages 1-3, while more *Allorhizobium-Neorhizobium-Pararhizobium-Rhizobium* and *Stenotrophomonas* taxa associated with nitrogen cycling and plant growth promotion were observed in stages 4-7. These findings have greatly improved our basic understanding of the assembly and functional adaptability of the endophytic microbiome during *A. muelleri* plant development and are helpful for the mining, development and utilization of functional microbial resources.

## 1 Introduction

Konjac species (*Amorphophallus* spp.) are unique in the world’s plant kingdom for their abundant content of konjac glucomannan (KGM). Due to its unique physical and chemical properties, KGM is widely used in food, medicine and health care, chemical industry, and other fields (Yang et al., 2024). At the same time, konjac is also an important economic crop widely planted in southwestern China and other regions (Yang et al., 2023; Li et al., 2023). *Amorphophallus muelleri*, an important cultivated species of *Amorphophallus*, has strong soft-rot disease resistance and rich KGM content. Most importantly, *A. muelleri* also has a high reproduction coefficient (Wei et al., 2022; Qi et al., 2023). After the flowering of *A. muelleri*, it can produce mature seeds without pollination (with the characteristics of triploid apomictic parthenogenesis). After maturity, 300-900 seeds can be obtained per spike, which expands the reproduction multiple of *Amorphophallus* from the traditional 4-6 times to 300-900 times. *A. muelleri* plants represent one of the triumphant examples of large-scale propagation that harnesses the power of triploid apomixis globally (Zhao et al., 2024). Presently, the seeds of *A. muelleri* have gained extensive traction and are extensively utilized in China (Yang et al., 2022). Therefore, as an important reproductive material for *A. muelleri*, it has important theoretical and practical significance to conduct in-depth research on seed growth and development.

The seeds of *A. muelleri* exhibit a more protracted growth and development cycle, coupled with an extended dormancy phase, in contrast to conventional corn and wheat seeds. Typically, these seeds necessitate a duration of approximately 8 months to reach maturity. Once mature, they enter a dormancy phase that can last for as long as 4 months before they are capable of germination and the emergence of new plants (Zhao et al., 2024). In previous research, we revealed the mechanisms of seed maturation in *A. muelleri* by analyzing endophytic microbial communities, transcriptomic, and metabolomic datasets (Zhao et al., 2024). Our results emphasized that the composition and function of the *A. muelleri* seed endophytic bacterial community are driven by the seed maturation state (Yang et al., 2022). However, the changes and functional adaptability of the endophytic microbial community in *A. muelleri* seeds during further dormancy breaking and new seedling establishment have not yet been explored.

All animal and plant species have complex associated microbial communities both on surfaces and in their interiors (Poupin et al., 2023). These microbes form a complex symbiotic relationship with plants and play an important role in promoting the productivity and health of plants in the natural environment (Turner et al., 2013; Vandenkoornhuyse et al., 2015; Hassani et al., 2018; Simon et al., 2019). Due to their location advantage, endophytic microbes are considered to have important effects on the growth and development of plants, for example, by fixing nitrogen, producing auxin, promoting plant nutrient acquisition, synthesizing antibacterial compounds, and enhancing plant tolerance to environmental stresses (Liu et al., 2017; Kumar et al., 2021). Some endophytes accompany plants throughout their life cycle, from seed germination to development, growth and fruiting (Truyens et al., 2015; Nelson, 2018). Understanding the assembly, functional adaptability and temporal dynamics of the plant endophytic microbiome at different developmental stages is important for the development of microbiome-based solutions for sustainable crop production systems (Singh et al., 2020; Xiong et al., 2021a; D’Hondt et al., 2021).

As a reproductive organ, seeds are so important in the life cycle of spermatophytes that they can remain dormant for long periods of time until the growing conditions are suitable for development into new plants (Ku niar et al., 2020). Seed endophytes represent a unique niche microbiota and are of special concern among seed-associated microbes (Nelson, 2018; Zhang C. M. et al., 2022). The seeds of many crops, such as rice, wheat, corn, and cotton, contain endophytes. Seed endophytes and fungi promote seed germination or seedling morphogenesis through the production of auxin, cytokinins, iron carriers, and the mobilization of various nutrients (N, P, K, etc.) and directly or indirectly improve the adaptability of developing plants by producing antibacterial compounds and inducing or regulating the expression of genes associated with plant growth, development and defense (Gond et al., 2015; Mousa et al., 2016; Irizarry and White, 2018). Seed-associated endophytic microbes play important roles in nutrient uptake and the reduction in abiotic and biotic stresses (Weyens et al., 2009; da Silveira et al., 2019). Recent studies have emphasized that the seed microbiota represents the starting point for the assembly of the microbial community in new seedlings, as well as the end point of the assembly of the community within the seed (Shade et al., 2017; Shahzad et al., 2018). The coevolution of seed core endophytic microbes with their hosts has resulted in a “continuation of the partnership” and led to the formation of a powerful and efficient transmission strategy between several generations (Truyens et al., 2015; Mousa et al., 2016; Frank et al., 2017). At different developmental stages, the host plant exerts a strong selection effect on its microbial communities through the host immune system, genetic network and plant secretions (Huang et al., 2019; Shakir et al., 2021). Meanwhile, the composition and potential functions of the plant microbiome also change dynamically during plant growth (Xiong et al., 2021a). However, at present, the connection between endophytic microbial communities in *A. muelleri* seeds and the host plant’s development is largely unexplored. Therefore, in-depth analyses of the composition of the core endophytic microbiota during seed development not only helps researchers to systematically understand the coevolution mechanisms of holozoans but also helps in the mining, development and utilization of functional microbial resources.

In this study, we hypothesized that different developmental stages drive the assembly and stability of the endophytic microbial community in *A. muelleri* seeds/seedlings. We used amplicon (16S and ITS) and metagenomic sequencing technologies to analyze the changes in the composition and function of microbiome communities during the process from seed germination to seedling establishment in *A. muelleri* (seven stages) and explored the temporal dynamics of microbial networks and the ecological functions of bacterial and fungal communities during plant developmental stages. Our study results elucidate the connection between endophytic microbial communities and host plant phylogeny, which will be helpful for the mining, development and utilization of functional microbial resources of *A. muelleri* seeds.

## 2 Materials and methods

### 2.1 Experiment description and sample collection

**FIGURE 1:**
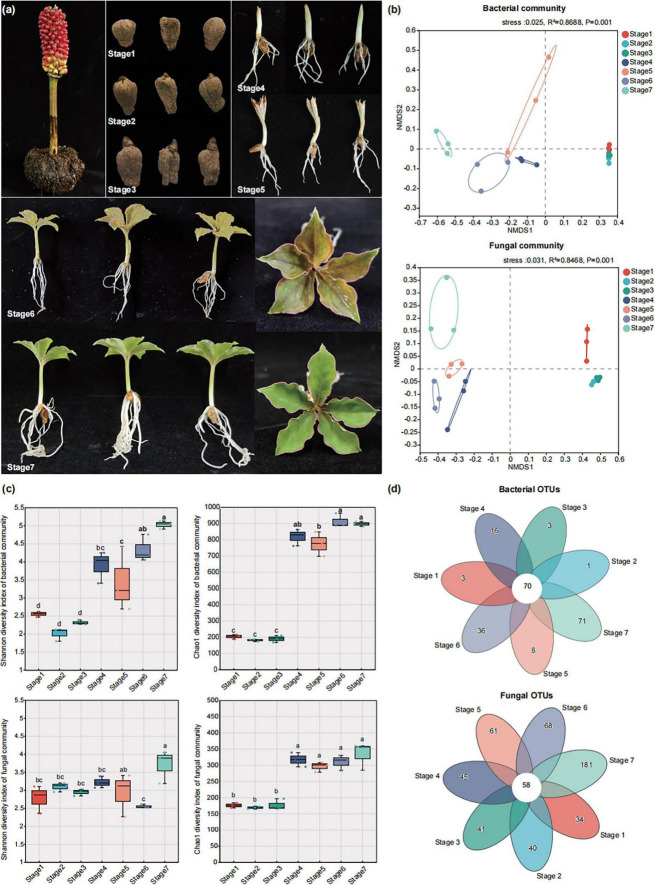
The seeds development of *A. muelleri* and factors that affect the assembly of endophytic microbial communities in its seeds and seedlings. **(a)** *A. muelleri* seeds maturation and different developmental stages. The mature seeds of *A. muelleri* turn completely red, then go through seven developmental stages to grow into new seedlings. Stage 1: the dormant period; Stage 2: the early shoot differentiation stage; Stage 3: the mid-shoot differentiation stage; Stage 4: the root tissue differentiation stage; Stage 5: the leaf differentiation stage; Stage 6: the early stage of leaf expansion. At this time, the leaves are mainly brown-green. Stage 7: the late stage of leaf expansion, the leaves have turned completely green. **(b)** Non-metric multi-dimensional scaling (NMDS) ordinations of Bray–Cutis dissimilarity matrices with permutational analysis of variance (PERMANOVA), showing the composition of bacterial (up) and fungal (down) communities at different developmental stages. **(c)** Shannon and Chao1 diversity indices of bacterial and fungal communities in different development stage of seeds and seedlings. **(d)** The number of unique, shared, and common bacterial and fungal operational taxonomic units at different groups.

The seeds of *A. muelleri* used in this study were provided by the Kunming University/Yunnan Key Laboratory of Konjac Biology. All the seeds had the same genetic background (variety: Zhuyajin1). In March 2023, *A. muelleri* seeds of the same size were selected and planted in plug trays filled with peat soil. All the plug trays were placed in plastic greenhouses at the Yunnan Key Laboratory of Konjac Biology, and watered by spraying 3-4 times a week without fertilization. According to tissue differentiation at different developmental stages, we collected *A. muelleri* samples at seven key time points, namely, the dormant period (Stage 1), the early bud differentiation stage (Stage 2), the mid-bud differentiation stage (Stage 3), the root tissue differentiation stage (Stage 4), the leaf differentiation stage (Stage 5), the early stage of leaf expansion (Stage 6), and the late stage of leaf expansion (Stage 7) (Figure 1a). The samples were collected between March and June 2023.

At each sampling stage, the entire konjac seeds/seedlings were removed from the peat soil and washed extensively with sterile water to remove any adhering soil from the seeds/seedlings. Then the seeds/seedlings materials were soaked with 75% ethanol for 30 s, washed with sterile water for 5 min, soaked in a 3% sodium hypochlorite solution for 3 min, and finally washed with sterile water 3 times, each time for 5 min. After surface sterilization, the entire konjac seeds and seedlings (including both roots and shoots) were cut into small pieces with an aseptic surgical knife in the ultra-clean workbench and were mixed thoroughly. The mixed samples were placed in 10 ml sterile conical tubes, quickly frozen in liquid nitrogen, and then stored immediately in a refrigerator at –80°C for future use. Seven treatments were implemented, with three replicates for each treatment, and each replicate contained three independent seeds/seedlings. Each replicate consisted of a composite sample obtained by mixing three individual seeds/seedlings.

### 2.2 Amplicon sequencing

#### 2.2.1 DNA extraction and Sequencing

Total DNA was extracted from the seeds and seedlings samples with the FastDNA^®^ Spin Kit for Soil (MP Biomedicals, Southern California, United States) according to the instructions. DNA concentration and purity were examined using a NanoDrop 2000, and DNA extraction quality was examined using 1% agarose gel electrophoresis. The endophytic fungal diversity was analyzed using the universal primers ITS1 F/ITS2 R. In the analysis of bacterial communities, a two-step PCR amplification was performed, with bacterial primers 799F/1392R and 799F/1193R used for 16S rRNA gene amplification, respectively. The PCR amplification conditions are shown in Supplementary Table 1 (Wang C. N. et al., 2021; Wang H. et al., 2021). The primer set 799F–1193R resulted in a lower co-amplification of chloroplast and mitochondrial genes, and more taxa covered the bacterial structure of the rhizosphere and endosphere (Wang et al., 2018). Amplicon libraries were sequenced on the Illumina HiSeq 2500 platform in Shanghai Majorbio Bio-Pharm Technology Co., Ltd.

#### 2.2.2 Analysis of amplicon sequencing data

The raw sequencing reads were quality-controlled using Fastp (v0.19.6) and then paired reads were merged into a single sequence using FLASH software (v1.2.11) (Magoçago, 2011). The processed high-quality sequencing reads were clustered into operational taxonomic units (OTUs) by UPARSE software (v11) (Edgar, 2013) based on a 97% similarity threshold. OTU taxonomic annotation was performed using SILVA (v138) (Quast et al., 2013) reference database and UNITE (v8.0) (Kõljalg et al., 2005) database for bacteria and fungi, respectively. Alpha diversity indices, including the Shannon and Chao 1 index, were calculated by Mothur software (v1.30.2). Wilcoxon rank sum test was used to analyze the differences in alpha diversity among different groups. Bray–Curtis dissimilarity matrices were calculated and visualized using non-metric multi-dimensional scaling (NMDS) ordinations to examine the overall changes in microbial community structure between samples, and the permutational multivariate analysis of variance (PERMANOVA) statistical tests were used to analyze whether the differences in microbial community structure between sample groups were significant (Oksanen et al., 2007). Co-occurrence network analysis of bacterial and fungal communities was conducted using the SparCC method (correlation coefficient > 0.75, *P* < 0.05) on the integrated network analysis pipeline (iNAP)^1^ (Feng et al., 2022). The networks were visualized using the interactive platform Gephi (Bastian et al., 2009). Nodes represent the individual microbial genera, and edges represent the pairwise correlations between the nodes in the microbiome network.

### 2.3 Metagenomic sequencing and data analysis

To further characterize the endophytic microbiome function within *A. muelleri* seeds/seedlings, metagenomic sequencing was performed on 21 DNA samples (7 stages × 3 replicates) using the Illumina NovaSeq platform with a paired-end protocol (Majorbio Bio-pharm Technology, Shanghai, China). The sequenced data were analyzed on the Majorbio Cloud Platform^2^ (Ren et al., 2022; Han et al., 2024). Specifically, raw sequences were quality-filtered using Fastp (v0.23.0) (Chen et al., 2018), and sequences belonging to *A. muelleri* genomes were removed by mapping the data to *A. muelleri* reference genomes using BWA (v0.7.17). The remaining reads were assembled using MEGAHIT (v1.1.2) (Li et al., 2015), with genes predicted from the resulting contigs using Prodigal (v2.6.3) (Hyatt et al., 2010). These genes were then clustered at 90% sequence identity and 90% coverage using CD-HIT (v4.6.1) (Fu et al., 2012) to generate a non-redundant gene catalog. High-quality reads were aligned to non-redundant gene catalogs to calculate gene abundance with 95% identity using SOAPaligner (Li et al., 2008). Representative sequences of the non-redundant gene catalog were aligned to the NR database (v20230830) with an *e*-value cutoff of 1e^–5^ using Diamond (v2.0.13) (Buchfink et al., 2015) for taxonomic annotations. The KEGG annotation and cluster of orthologous groups of proteins (COG) annotation for the representative sequences were performed using Diamond (v2.0.13) against the Kyoto Encyclopedia of Genes and Genomes database (v20230830) and COG database (v2020), with an *e*-value cutoff of 1e^–5^. Carbohydrate-active enzyme annotation was conducted using hmmscan against the CAZy database (v12) with an *e*-value cutoff of 1e^–5^. A nonparametric statistical test (Kruskal-Wallis test) was performed to evaluate differences in functions or gene abundance among different groups.

## 3 Results and analysis

### 3.1 Diversity and community composition of the microbial communities in *Amorphophallus muelleri* at different developmental stages

A total of 12,04,705 effective bacterial sequences and 10,94,749 effective fungal sequences were obtained from 21 plant samples, with average lengths of 376 and 233 bp, respectively. Taxonomic annotation was performed on the valid reads at the 97% sequence similarity level, and a total of 1,207 bacterial and 1,039 fungal operational taxa were recovered from all 21 samples. Non-metric multidimensional scaling (NMDS) ordination and permutational multivariate ANOVA (PERMANOVA) showed that plant developmental stages explained greater changes in the bacterial and fungal communities, especially the microbial community compositions before rooting and after rooting, which were significantly different (*R*^2^ = 0.869 for bacteria and *R*^2^ = 0.847 for fungi, *P* = < 0.001 for both) (Figure 1b). Analysis of the diversity and richness of the bacterial and fungal communities revealed that the plant developmental stage also affected the diversity of the microbial community and that the diversity and richness of the microbial communities in the *A. muelleri* tissues increased as the seeds germinated (Figure 1c). After seminal root tissue differentiation (Stage 4, Stage 5, Stage 6 and Stage 7), the Shannon index and Chao1 index values for the bacterial community in *A. muelleri* tissue were both greater than those before rooting (Stage 1, Stage 2 and Stage 3) (*P* < 0.05), and the diversity and richness of the bacterial communities was the highest in the Stage 7 group. Similarly to that in the bacterial community, the Chao1 index values for the fungal communities in the Stage 4, Stage 5, Stage 6, and Stage 7 groups were significantly higher than those in the Stage 1, Stage 2, and Stage 3 groups (*P* < 0.05); the diversity and richness of the fungal communities were also highest in the Stage 7 group. No significant differences were found in the Shannon and Chao1 indices of the direct bacterial and fungal communities among the Stage 1, Stage 2, and Stage 3 groups (*P* > 0.05).

A Venn diagram further demonstrated the effect of plant developmental stage on the composition of the bacterial and fungal communities. We identified 70 core bacterial taxa and 58 core fungal taxa that coexisted in *A. muelleri* tissue samples at different developmental stages (Figure 1d). We noted that the Stage 7 group had the greatest number of unique bacterial and fungal OTUs, followed by the Stage 6 group.

### 3.2 Species composition of the microbial communities in *Amorphophallus muelleri* at different developmental stages

Next, we analyzed the species composition at different developmental stages at different taxonomic levels. At the phylum level, before rooting (Stage 1, Stage 2, and Stage 3), Proteobacteria, Bacteroidota, and Firmicutes were the most dominant bacterial phyla in the konjac tissues, and Ascomycota and Basidiomycota were the most dominant fungal phyla (Supplementary Figures 1a,b). Among them, the relative abundance of Proteobacteria in the Stage 2 (83.79%) samples was significantly higher than that in the Stage 1 (71.64%) and Stage 3 (73.14%) samples (*P* < 0.05) (Supplementary Table 2); the relative abundance of Basidiomycota in the Stage 2 (9.78%) and Stage 3 (10.76%) samples was significantly higher than that in the Stage 1 (3.07%) samples (P < 0.05) (Supplementary Table 3). In contrast, after rooting (Stage 4, Stage 5, Stage 6, and Stage 7), Proteobacteria, Bacteroidota, and Actinobacteria were the most dominant bacterial phyla, and Ascomycota, Rozellomycota and Basidiomycota were the most dominant fungal phyla (Supplementary Figures 1a,b). Compared with the three treatments before root emergence, we observed that the relative abundances of Firmicutes and Ascomycota in the four treatments after root emergence were significantly decreased, while the relative abundance of Rozellomycota was significantly increased (*P* < 0.05) (Supplementary Tables 2, 3).

**FIGURE 2:**
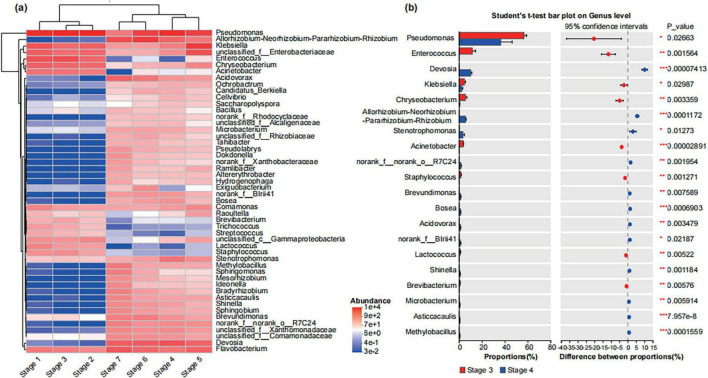
Composition of bacterial community in different developmental stages of seeds and seedlings at the genus level. **(a)** The community heatmap shows the composition of the top 50 with relatively rich bacterial community genera in different groups. Here: The abundance changes of different species in the samples are displayed through the color gradient of the color blocks, with the gradient bar on the right of the figure representing the numerical values corresponding to the color gradient. **(b)** The Student’s *t*-test shows the differences in the average relative abundances of the same bacterial genus between the Stage 3 and Stage 4. Here: *Stands for *P* < 0.05, **stands for *P* < 0.01, and ***stands for *P* < 0.001.

**FIGURE 3:**
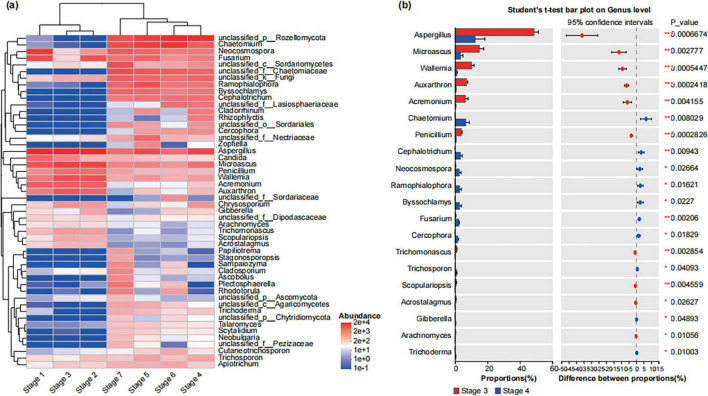
Composition of fungal community in different developmental stages of seeds and seedlings at the genus level. **(a)** The community heatmap shows the composition of the top 50 with relatively rich fungal community genera in different groups. Here: The abundance changes of different species in the samples are displayed through the color gradient of the color blocks, with the gradient bar on the right of the figure representing the numerical values corresponding to the color gradient. **(b)** The Student’s *t*-test shows the differences in the average relative abundances of the same fungal genus between the Stage 3 and Stage 4. Here: *Stands for *P* < 0.05, **stands for *P* < 0.01, and ***stands for *P* < 0.001.

We used a community heatmap to show the species composition of the top 50 abundant species in the bacterial and fungal communities at the genus level (Figures 2, 3). The samples from different treatment groups were primarily divided into two major branches. The samples from the before rooting stage (Stage 1, Stage 2, and Stage 3) clustered together, while those from the after rooting stage (Stage 4, Stage 5, Stage 6, and Stage 7) formed another cluster, further suggesting the differences in the composition of bacterial and fungal communities in *A. muelleri* tissues before and after rooting (Figures 2a, 3a). Among the top 50 bacterial genera in relative abundance, the relative abundance of *Pseudomonas* in the samples before rooting was higher than that in the samples after rooting. With increasing development time, the relative abundance of *Pseudomonas* gradually decreased from Stage 4 to Stage 7, with the relative abundance being the lowest in the Stage 7 samples. *Acidovorax* and *Devosia* species were significantly enriched in all samples after rooting (Stage 4, Stage 5, Stage 6, and Stage 7) (*P* < 0.05), while *Flavobacterium* species were significantly enriched in samples after leaf development (Stage 5, Stage 6, and Stage 7) (*P* < 0.05) (Supplementary Figure 2). Among the top 50 fungal genera in relative abundance, compared with those in the Stage 1 samples, the abundances of *Neocosmospora* and *Fusarium* were significantly lower in the Stage 2 and Stage 3 samples (*P* < 0.05), and the abundance of *Candida* was also significantly lower in the samples at different developmental stages (*P* < 0.05) (Supplementary Figure 3). Species of the genera *Chaetomium, Ramophialophora, Byssochlamys, Cephalotrichum*, and *Cercophora* were enriched in all samples after rooting (Stage 4 to Stage 7) compared to samples before rooting. The relative abundances of *Papiliotrema, Stagonosporopsis, Sampaiozyma, Cladosporium, Ascobolus, Plectosphaerella*, and *Rhodotorula* were the highest in the Stage 7 samples (Figure 3a).

We further analyzed the differences in bacterial and fungal communities between Stage 3 and Stage 4. The results showed that the relative abundances of bacterial genera such as *Pseudomonas*, *Enterococcus*, *Klebsiella*, *Chryseobacterium*, *Acinetobacter* and fungal genera such as *Aspergillus*, *Microascus*, *Wallemia*, *Auxarthron*, *Acremonium*, *Penicillium* in Stage 4 samples were significantly lower than those in Stage 3 samples (*P* < 0.05) (Figures 2b, 3b); conversely, the relative abundances of bacterial genera such as *Devosia*, *Allorhizobium- Neorhizobium-Pararhizobium-Rhizobium*, *Stenotrophomonas* and fungal genera such as *Chaetomium*, *Cephalotrichum*, *Neocosmospora*, *Ramophialophora*, *Byssochlamys*, *Fusarium* were significantly increased compared with those in Stage 3 (*P* < 0.05) (Figures 2b, 3b).

### 3.3 Co-occurrence network of microbial communities in *Amorphophallus muelleri* at different developmental stages

**FIGURE 4:**
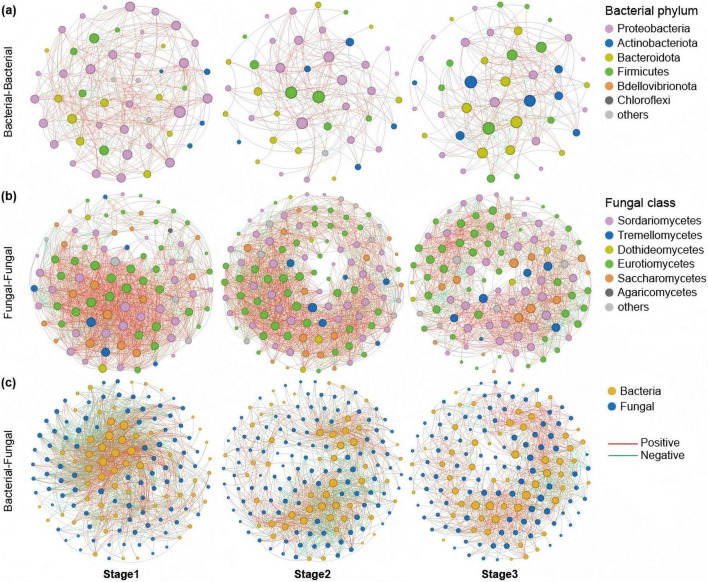
Co-occurrence network of microbial communities in three stages before tissue differentiation in the root (Stage 1, Stage 2, and Stage 3). **(a)** The bacterial intradomain networks show a lower number of nodes and edges at Stage 2 and Stage 3 compared to Stage 1. **(b)** The fungal intradomain networks show more nodes and edges in Stage 2 compared to Stage 1. However, there are more nodes in Stage 3 than in Stage 1, but with fewer edges. **(c)** Interdomain networks between bacteria and fungi show a higher number of nodes and edges at Stage 2 compared to Stage 1 and Stage 3. The interdomain network correlation was mainly negative in Stage 1, and the interdomain correlation in Stage 3 and Stage 2 was mainly positive. The nodes are colored based on bacterial phyla and fungal classes, and edge color represents positive (red) and negative (green) correlations.

**FIGURE 5:**
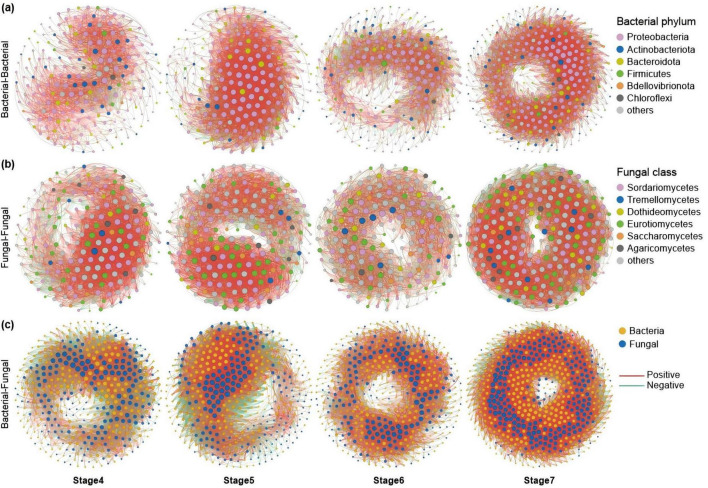
Co-occurrence network of microbial communities in four stages after tissue differentiation in the root (Stage 4, Stage 5, Stage 6, and Stage 7). **(a)** The bacterial intradomain networks show a higher number of nodes and edges at Stage 5, Stage 6, and Stage 7 compared to Stage 4. **(b)** The fungal intradomain networks show the number of nodes and edges in Stage 7 is higher than in the other three stages. **(c)** Interdomain networks between bacteria and fungi show that Stage 7 has the highest number of nodes and edges among the four stages, and there are more edges in Stage 5 and Stage 6 than in Stage 4. The correlations of the interdomain network in Stage 4 and Stage 5 were dominated by negative correlations. During Stage 6 and Stage 7, the interdomain networks tended to be stable, and there was no significant difference in the proportion of positive and negative correlations (approximately 50%, respectively).

To study the co-occurrence patterns of *A. muelleri* microbial communities at different developmental stages, we analyzed the bacteria-bacteria and fungi-fungi intrakingdom networks and bacteria-fungi interkingdom network in *A. muelleri* at different developmental stages (Figures 4, 5). Intradomain network analysis revealed that bacteria and fungi played different network roles during the development of *A. muelleri*. During the seven developmental stages, the fungal taxa always had higher network connectivity than the bacterial taxa (i.e., average degree, bacteria: 7.455-49.605; fungi: 22.296-75.5) (Supplementary Table 4). In the *A. muelleri* tissues, before rooting (Stages 1–3), the complexity of the bacterial network was lower than that of the fungal network, and both the number of nodes and edges in the fungal network were higher than those in the bacterial network (Figures 4a,b and Supplementary Table 4). After root growth, the number of nodes and edges and the average degree of the bacterial network increased compared with those before rooting. Compared with the fungal network, the bacterial network had more nodes but a lower average connectivity (Figures 5a,b and Supplementary Table 4). During different developmental stages, network correlations within both the bacterial and fungal intrakingdom networks were mainly positive (Figures 4a,b, 5a,b and Supplementary Table 4).

Similarly, the ecological network analysis between the bacteria and the fungi showed that the number of nodes and edges in the interdomain network before rooting was lower than that after rooting. After the roots grew, both the number of nodes and edges and the average degree of the interdomain network increased, with the highest number of nodes and edges observed at stage 7 (Figures 4c, 5c). The interdomain network correlation was mainly negative in the Stage 1 group, and the interdomain correlation in the Stage 2 and Stage 3 groups was mainly positive (Figure 4c and Supplementary Table 4). The correlations of the interdomain network in the Stage 4 and Stage 5 groups were mainly negative; in the Stage 6 and Stage 7 groups, the interdomain networks tended to be stable, and there was no significant difference in the proportion of positive and negative correlations (close to 50%, respectively) (Figure 5c and Supplementary Table 4).

### 3.4 Functional composition of microbial communities in *Amorphophallus muelleri* at different developmental stages

**FIGURE 6:**
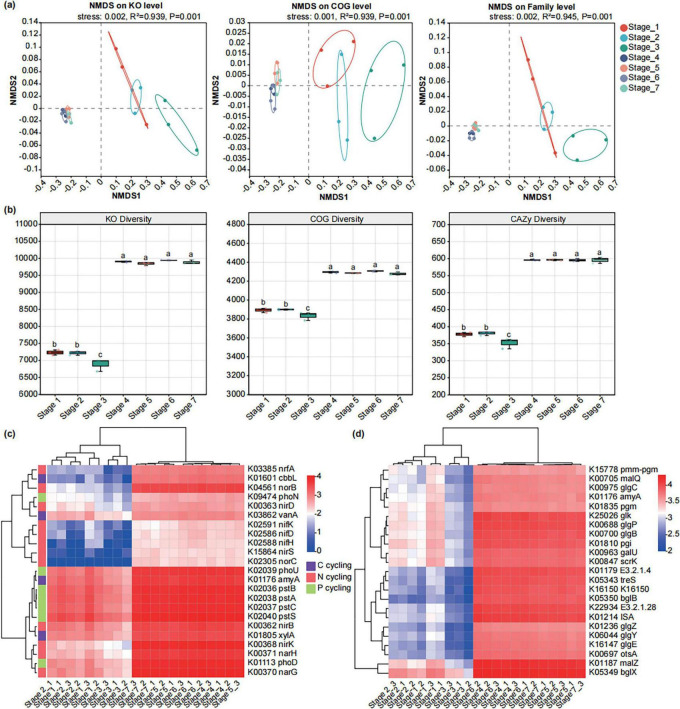
Functional profiles of microbiomes in different developmental stages of seeds and seedlings. **(a)** NMDS ordinations of functional genes based on Bray-Curtis distance matrices of KO, COG, and CAZy functional genes show the distinct microbial communities’ function in seeds and seedlings at different developmental stages. **(b)** The boxplot shows the functional diversity (including KO, COG, and CAZy) of the microbiomes of seeds and seedlings across seven developmental stages. **(c)** Heatmap exhibiting the relative abundance of functional genes (based on KO) involved in C, N, and P cycling which varied among seven developmental stages. **(d)** Heatmap exhibiting the relative abundance of functional genes (based on KO) involved in starch and sucrose metabolism which varied among seven developmental stages.

We used metagenomic sequencing to explore the functional transformation of *A. muelleri*-associated microbial communities at different developmental stages. We performed metagenomic sequencing on 21 samples. Following quality control, the 21 samples yielded an average of approximately 87,989,508 clean reads. After filtering out host-derived sequences, we retained an average of 53,473,380 high-quality reads, which were subsequently assembled into an average of 606,730 contigs (Supplementary Table 5). NMDS ordination analysis revealed that the functional composition of the microbiome (i.e., KO, eggNOG, and CAZy) was affected by the *A. muelleri* developmental stages. In particular, there were significant differences in the functional composition of the microbiome before and after rooting (*R*^2^: KO = 0.939, eggNOG = 0.939, CAZy = 0.945; *P* < 0.01) (Figure 6a). In addition to the functional composition, the functional diversity of the endophytic microbiome of *A. muelleri* was also affected by developmental stage. Compared with before rooting, the four stages after rooting had higher microbiome functional diversity (i.e., the richness of Chao1 based on KO, eggNOG, and CAZy), but no significant difference was observed among the four stages. In the three stages before rooting, the functional diversity of the endophytic microbiota in Stage 3 was significantly lower than that in Stage 1 and Stage 2 (*P* < 0.05) (Figure 6b).

To determine the functional characteristics of the microbiomes of endophytes of *A. muelleri* at different developmental stages, we performed differential abundance analysis. Some carbon, nitrogen, and phosphorus cycle- and sucrose and starch metabolism-related genes exhibited different abundance patterns at different developmental stages (Figure 6c). Compared with those before and after rooting, carbon, nitrogen and phosphorus cycle-related genes were more abundant in the four stages after rooting. Among the three stages before rooting, Stage 3 generally showed lower relative abundance of genes related to carbon, nitrogen, and phosphorus cycling compared to Stage 1 and Stage 2. Similarly, genes associated with sucrose and starch metabolism were more abundant across the four stages after rooting, with Stage 3 exhibiting the lowest relative abundance (Figure 6d).

**FIGURE 7:**
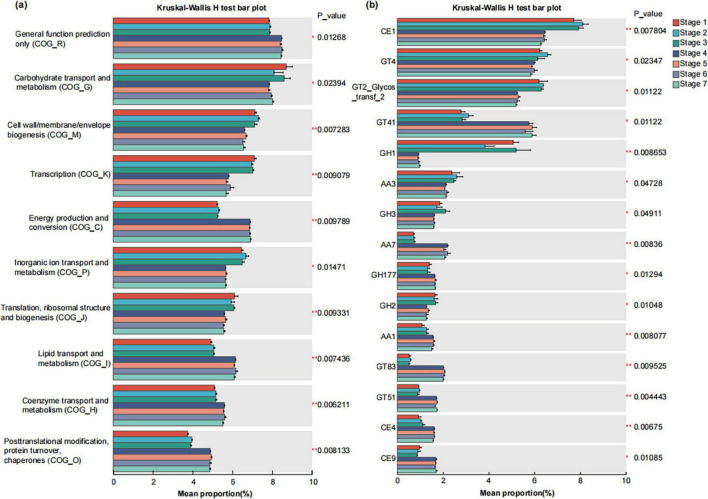
Differential abundance analysis of COG **(a)** and CAZy **(b)** functional genes of the microbiomes of seeds and seedlings across seven developmental stages. The vertical axis represents the gene name, the horizontal a certain gene abundance in the horizontal axis represents the percentage of a certain gene abundance in the sample, and different colors represent different groups. *p* < = 0.05*, 0.001, < *p* < = 0.01**.

In the COG functional categories, we observed that genes related to carbohydrate transport and metabolism (COG_G), cell wall/membrane/envelope biogenesis (COG_M), transcription (COG_K), inorganic ion transport and metabolism (COG_P), and translation, ribosomal structure, and biogenesis (COG_J) exhibited higher relative abundance in the three stages before rooting. In contrast, genes associated with energy production and conversion (COG_C), lipid transport and metabolism (COG_I), coenzyme transport and metabolism (COG_H), and posttranslational modification, protein turnover, chaperones (COG_O) showed higher relative abundance in the four stages after rooting. Notably, among all seven stages, cell wall/membrane/envelope biogenesis (COG_M) and inorganic ion transport and metabolism (COG_P) reached their highest relative abundance in Stage 2 (Figure 7a).

The bar chart shows the top 15 CAZy functions (based on Family) in relative abundance that have significant differences among different groups. The relative abundances of CE1 family, GH1 family, GH2 family, and AA3 family were significantly higher in the three stages before rooting than in the four stages after rooting (*P* < 0.05). In contrast, the relative abundances of GT41 family, AA7 family, GH177 family, GT83 family, GT51 family, CE4 family, and CE9 family were significantly lower in the before rooting stages compared to the after rooting stages (*P* < 0.05). Among all seven stages, the CE1 family and GT4 family reached their highest relative abundance in Stage 2 (Figure 7b).

## 4 Discussion

### 4.1 Effect of developmental stage on the assembly of endophytic microbiota in *Amorphophallus muelleri*

The assembly of the plant microbiome starts shortly after sowing and continues to evolve as plants grow and develop (Bulgarelli et al., 2013; Müller et al., 2016). In addition to the genetic and vertical transmission of microorganisms from seeds (Müller et al., 2016; Abdelfattah et al., 2021), microbes can also colonize different plant compartments through soil, air, and the diffusion of nearby plants and then form dynamic communities under the comprehensive action of host and environmental factors (Vandenkoornhuyse et al., 2015; Vorholt et al., 2017; Hassani et al., 2018; Compant et al., 2021). The healthy growth of plants depends on homeostasis, which is largely maintained by three factors: the environment, host genetics and the microbiome (Trivedi et al., 2020). The elucidation of the ecological principles and processes underpinning the assembly and developmental dynamics of plant microbiomes is critical for advancing the basic understanding of coevolution and the application of crop microbiomes in the future sustainable improvements in agricultural productivity (Singh and Trivedi, 2017; Sessitsch et al., 2019; Singh et al., 2020; Trivedi et al., 2020).

Herein, we first evaluated the effects of seven developmental stages on the assembly of the endophytic microbiota of *A. muelleri*. The results showed that plant developmental stage explained the large variation in the endophytic bacterial and fungal communities of *A. muelleri* and that a variety of microbial attributes (such as α-diversity, β-diversity, community composition, and the ecological network of bacteria and fungi) were all driven by the developmental status of *A. muelleri*. The NMDS ordination results showed that the differentiation and formation of *A. muelleri* tissues (especially root development and differentiation) significantly affected the assembly of the endophytic microbiota of *A. muelleri* in the three stages before rooting and in the four stages after rooting, with a clear difference in distribution along the NMDS1 axis, indicating that the endophytic microbial community structure of *A. muelleri* significantly differed before and after rooting. In addition, compared with those in the stages before rooting, the bacterial and fungal communities in the four stages after rooting had higher diversity and richness, while the microbial community composition and diversity in the Stage 1, Stage 2, and Stage 3 before rooting were not significantly different. The Heatmap of the bacterial and fungal communities at the genus level shows that the samples before and after rooting are divided into two distinct branches, further suggesting that root development and differentiation have a significant impact on the composition of endophytic microbiota in *A. muelleri*. Based on these results, we speculate that soil may be one of the important sources of endophytic microbiota in *A. muelleri*. These findings are consistent with previous studies, which indicated that the influences of plant developmental stages on the microbiome include the influence of environmental factors; soil, air, rainwater, etc., are also important sources of the endophytic microbiome (Lindow and Brandl, 2003; Vacher et al., 2016; Xiong et al., 2021a).

Studies have shown that the composition and potential function of the plant microbiome dynamically change during plant growth (Conn et al., 2008; Lee et al., 2021; Xiong et al., 2021a), but some core microbes involved in seed dispersal coevolved with their hosts and are present throughout the entire life cycle of the plant (Truyens et al., 2015; Zhang C. M. et al., 2022). Based on a limited understanding of the plant microbiome, it has been proposed that the dynamics of plant microbiome composition reflect the current needs of the host plant (Coyte et al., 2015; Fitzpatrick et al., 2020) and represent the result of subtle changes in the microbial selection strategy imposed by the host during plant development (Martin et al., 2017; Morella et al., 2019; Finkel et al., 2020; Xiong et al., 2021b). In this dynamic process, host plants mainly use “central microbes” to regulate the interactions between microbes and change host fitness to selectively affect the structure of their associated microbiota (Agler et al., 2016; Toju et al., 2018; Roman-Reyn et al., 2019). In this study, we found that 70 and 58 core bacterial and fungal taxa coexisted in *A. muelleri* tissue samples at different developmental stages. From seed germination to tissue differentiation, the number of OTUs unique to different developmental stages of *A. muelleri* showed an increasing trend, with the last completely green leaf stage (Stage 7) having the largest number of bacterial and fungal OTUs and the highest microbial diversity and richness. The microbial network analysis results showed that bacteria and fungi play different network roles during *A. muelleri* seed development. During the seven developmental stages, fungal taxa always had higher network connectivity than did bacterial taxa, especially before rooting (Stage 1, Stage 2, and Stage 3). The number of nodes and edges in the fungal network in *A. muelleri* tissues was higher than that in the bacterial network, suggesting the ecological importance of fungal taxa in *A. muelleri* seed development. We further found that the proportion of positive edges, representing the bacteria-fungi interdomain correlation, gradually increased from Stage 1 to Stage 2 and then to Stage 3. A positive interaction meant that the competition between bacteria and fungi weakened. In the Stage 4, the development of the *A. muelleri* root tissue was complete, and a large number of exogenous microbes may have entered the plant body through the roots. At this stage, the microbial communities of *A. muelleri* began to show large differences, and the proportion of negative edges in the bacteria-fungus interdomain correlation reached 69.0%. The competition among bacteria and fungi has increased significantly. Studies have shown that mutual negative interactions (i.e., ecological competition) can improve the stability of microbial communities by inhibiting the destabilizing effect of cooperation (Coyte et al., 2015; Santolini and Barabasi, 2018). A host may benefit from microbe competition, thereby improving resistance to external stress (Wagg et al., 2019). Our results also showed that with increasing developmental stage of *A. muelleri*, the proportion of negative edges between bacteria and fungi in Stage 4 to Stage 7 gradually decreased, and the interdomain network gradually stabilized. In conclusion, our research results provide important evidence for the assembly of the endophytic microbiome of *A. muelleri* driven by the plant developmental stage.

### 4.2 Composition and functional adaptation of endophytic microbiota in *Amorphophallus muelleri* at different developmental stages

The ecological functions of plants are implemented through the synergy of the plant microbiota. Our analyses revealed distinct functional profiles (KO/COG/CAZy) between the three stages before rooting and the four stages after rooting samples, demonstrating clear segregation along the NMDS1 axis (*P* < 0.01). This indicates significant functional divergence in endophytic microbial communities before versus after rooting. Compared with the stages before rooting, the four stages after rooting had higher functional diversity of microorganisms. Microbial functional genes involved in carbohydrate transport and metabolism; cell wall/membrane/envelope biogenesis; transcription; inorganic ion transport and metabolism; translation, and ribosome structure and biogenesis were abundant in *A. muelleri* seeds from Stage 1 to Stage 3. From Stage 4 to Stage 7, microbial functional genes involved in the carbon, nitrogen and phosphorus cycles, starch and sucrose metabolism, energy production and conversion, lipid transport and metabolism, and coenzyme transport and metabolism were more abundant. These changes further provided important evidence that the function of the microbial community is closely related to the developmental stage of *A. muelleri* seeds.

In the initial stage of seed germination, seeds mainly rely on stored organic substances (such as starch, fat, protein) as substrates for respiration to provide energy and intermediate products, so as to support growth activities such as cell division and differentiation. The decomposition and oxidation of these stored substances constitute important components of the carbon cycle (Weitbrecht et al., 2011). The results of our metagenomic sequencing revealed that C degradation-related genes, such as alpha-amylase (amyA) and vanillate monooxygenase (vanA) (Xiong et al., 2021a), exhibited higher relative abundance in the Stage 1 (pre-germination) and Stage 2 (early germination) compared to Stage 3. Furthermore, we observed significant enrichment of Proteobacteria and Basidiomycota taxa specifically during Stage 2. Proteobacteria can utilize refractory C sources by secreting hydrolytic enzymes (Verzeaux et al., 2016; Wei et al., 2018). Basidiomycota are also important decomposers in the carbon cycle, and they can secrete digestive enzymes to decompose organic substances (such as cellulose, lignocellulose) into smaller molecules (Sharma-Poudyal et al., 2017). Therefore, we hypothesize that the significant enrichment of Proteobacteria and Basidiomycota during Stage 2 may be linked to their involvement in carbon cycling, thereby providing energy for seed development. Our results also revealed that the C-fixation-related gene ribulose-bisphosphate carboxylase large chain (*cbbL*) was significantly enriched at the four stages from Stage 4 to Stage 7. Starting from Stage 4, plant photosynthesis is enhanced, and C fixation-related genes are significantly enriched, which helps plants fix atmospheric carbon dioxide, providing energy and carbon sources for plant growth and metabolism. Research has demonstrated that the photosynthesis process in quinoa becomes significantly more active following the stage of hypocotyl elongation during seed germination (Hao et al., 2022).

In addition, we also found that after the formation of *A. muelleri* root tissue, the functional genes of microorganisms participating in the N cycle were significantly enriched in the four stages from Stage 4 to Stage 7 in *A. muelleri*; additionally, their relative abundance was significantly greater than that in the three stages before rooting. When root and leaf tissues differentiate, the N cycle within plants usually intensifies. The N cycle plays an indispensable role in plant development by influencing nitrogen uptake and transport, promoting plant protein synthesis, supporting leaf growth, and facilitating plant hormone biosynthesis (Zayed et al., 2023). Coincidentally, microbial taxa related to the N cycle, such as *Allorhizobium-Neorhizobium-Pararhizobium-Rhizobium* and *Stenotrophomonas*, are also enriched to varying degrees at the stage after rooting. The significant enrichment of microorganisms and functional genes involved in the N cycle after the differentiation of *A. muelleri* root tissue further reflects the functional adaptability of endophytic microorganisms in *A. muelleri*.

Phosphorus cycling and transformation are also very important in the process of seed germination. Phosphorus is a component of biomolecules such as nucleic acids, proteins and phospholipids and plays a key role in seed cell division, DNA replication, protein synthesis and cell wall synthesis. Phosphorus also participates in the synthesis and degradation of ATP molecules and provides the necessary energy to support seed germination and growth (Malhotra et al., 2018; Khan et al., 2023). In this study, the relative abundance of phosphorus cycling-related genes (pstA, pstB, pstC, pstS, and phoU) in Stage 1 and Stage 2 samples was higher than that in Stage 3. Meanwhile, we observed that the relative abundance of *Pseudomonas* was higher in the samples of Stage 1, Stage 2, and Stage 3, but significantly decreased in the samples after Stage 4. As one of the dominant bacterial genera in common habitats, *Pseudomonas* is widely present in soil and within animals and plants. It is an important decomposer in the P cycle (Oteino et al., 2015; Anuroopa et al., 2021). Moreover, many strains within this genus are plant growth-promoting rhizobacteria (PGPR), and these strains colonize the plant rhizosphere and help plants resist diseases and promote plant growth (Zhang X. et al., 2022). Wen et al. (2023) reported that bacterial suspensions of the salt-tolerant bacteria *Pseudomonas koreensis* and *P. veronii* from rhizosphere saline soil promoted the growth of radicles and germination of oilseed rape. Whether the *Pseudomonas* species in this study is related to the promotion of *A. muelleri* seed germination remains to be further studied.

## 5 Conclusion

By studying the temporal dynamics of the endophytic bacterial and fungal communities of *A. muelleri* seeds during the period from germination to seedling establishment (seven stages), we have provided a systematic understanding of the assembly and functional adaptation of the endophytic microbiota during the development of *A. muelleri*. Our results showed that plant developmental stage explained the large variation in endophytic bacterial and fungal communities in *A. muelleri* and that multiple microbial attributes (e.g., α-diversity, β-diversity, community composition, and bacterial and fungal ecological networks) are driven by the developmental state of *A. muelleri*. These results further confirm that during the process from germination to seedling establishment, the changes in the composition and potential functional dynamics of the plant endophytic microbiota play an important role in promoting plant development and nutrient cycling. These findings have greatly improved our basic understanding of the connection between endophytic microbial communities and the developmental stage of host plants; have helped the mining, development, and utilization of functional microbial resources; and are important for the development of microbiome-based solutions for sustainable crop production systems.

## Data Availability

The datasets presented in this study can be found in online repositories. The names of the repository/repositories and accession number(s) can be found at: https://www.ncbi.nlm.nih.gov/, PRJNA1157566; https://www.ncbi.nlm.nih.gov/, PRJNA1158523.

## Footnotes

1
https://github.com/yedeng-lab/iNAP

2
www.majorbio.com

## References

[B1] Abdelfattah A. Wisniewski M. Schena L. Tack A. J. M. (2021). Experimental evidence of microbial inheritance in plants and transmission routes from seed to phyllosphere and root. *Environ. Microbiol.* 23 2199–2214. 10.1111/1462-2920.15392 33427409

[B2] Agler M. T. Ruhe J. Kroll S. Morhenn C. Kim S. T. Weigel D. (2016). Microbial hub taxa link host and abiotic factors to plant microbiome variation. *PLoS Biol.* 14:e1002352. 10.1371/journal.pbio.1002352 26788878 PMC4720289

[B3] Anuroopa N. Anshu B. R. Ranadev P. Ashwin R. Bagyaraj D. J. (2021). *Pseudomonas* species in soil as a natural resource for plant growth promotion and biocontrol characteristics-an overview. *Madras Agric. J.* 108. 10.29321/MAJ.10.000571 [Epub ahead of print].35428308

[B4] Bastian M. Heymann S. Jacomy M. (2009). Gephi: An open source software for exploring and manipulating networks. *ICWSM* 3 361–362. 10.1609/icwsm.v3i1.13937

[B5] Buchfink B. Xie C. Huson D. H. (2015). Fast and sensitive protein alignment using DIAMOND. *Nat. Methods* 12 59–60. 10.1038/nmeth.3176 25402007

[B6] Bulgarelli D. Schlaeppi K. Spaepen S. Ver Loren van Themaat E. Schulze-Lefert P. (2013). Structure and functions of the bacterial microbiota of plants. *Annu. Rev. Plant. Biol.* 64 807–838. 10.1146/annurev-arplant-050312-120106 23373698

[B7] Chen S. Zhou Y. Chen Y. Gu J. (2018). Fastp: An ultra-fast all-in-one FASTQ preprocessor. *Bioinformatics* 34 i884–i890. 10.1093/bioinformatics/bty560 30423086 PMC6129281

[B8] Compant S. Cambon M. C. Vacher C. Mitter B. Samad A. Sessitsch A. (2021). The plant endosphere world - bacterial life within plants. *Environ. Microbiol.* 23 1812–1829. 10.1111/1462-2920.15240 32955144

[B9] Conn V. M. Walker A. R. Franco C. M. (2008). Endophytic actinobacteria induce defense pathways in *Arabidopsis thaliana*. *Mol. Plant Microbe Interact.* 21 208–218. 10.1094/MPMI-21-2-0208 18184065

[B10] Coyte K. Z. Schluter J. Foster K. R. (2015). The ecology of the microbiome: Networks, competition, and stability. *Science* 350 663–666. 10.1126/science.aad2602 26542567

[B11] da Silveira A. P. D. Iório R. P. F. Marcos F. C. C. Fernandes A. O. de Souza S. A. C. D. Kuramae E. E. (2019). Exploitation of new endophytic bacteria and their ability to promote sugarcane growth and nitrogen nutrition. *Antonie Van Leeuwenhoek* 112 283–295. 10.1007/s10482-018-1157-y 30194506

[B12] D’Hondt K. Kostic T. McDowell R. Eudes F. Singh B. K. Sarkar S. (2021). Microbiome innovations for a sustainable future. *Nat. Microbiol.* 6 138–142. 10.1038/s41564-020-00857-w 33510435

[B13] Edgar R. C. (2013). UPARSE: Highly accurate OTU sequences from microbial amplicon reads. *Nat. Methods* 10 996–998. 10.1038/nmeth.2604 23955772

[B14] Feng K. Peng X. Zhang Z. Gu S. He Q. Shen W. (2022). iNAP: An integrated network analysis pipeline for microbiome studies. *Imeta* 1:e13. 10.1002/imt2.13 38868563 PMC10989900

[B15] Finkel O. M. Salas-González I. Castrillo G. Conway J. M. Law T. F. Teixeira P. J. P. L. (2020). A single bacterial genus maintains root growth in a complex microbiome. *Nature* 587 103–108. 10.1038/s41586-020-2778-7 32999461 PMC10329457

[B16] Fitzpatrick C. R. Salas-González I. Conway J. M. Finkel O. M. Gilbert S. Russ D. (2020). The plant microbiome: From ecology to reductionism and beyond. *Annu. Rev. Microbiol.* 74 81–100. 10.1146/annurev-micro-022620-014327 32530732

[B17] Frank A. C. Saldierna Guzmán J. P. Shay J. E. (2017). Transmission of bacterial endophytes. *Microorganisms* 5:70. 10.3390/microorganisms5040070 29125552 PMC5748579

[B18] Fu L. Niu B. Zhu Z. Wu S. Li W. (2012). CD-HIT: Accelerated for clustering the next-generation sequencing data. *Bioinformatics* 28 3150–3152. 10.1093/bioinformatics/bts565 23060610 PMC3516142

[B19] Gond S. K. Bergen M. S. Torres M. S. White J. F. (2015). Endophytic *Bacillus spp*. produce antifungal lipopeptides and induce host defence gene expression in maize. *Microbiol. Res.* 172 79–87. 10.1016/j.micres.2014.11.004 25497916

[B20] Han C. Shi C. Liu L. Han J. Yang Q. Wang Y. (2024). Majorbio cloud 2024: Update single-cell and multiomics workflows. *Imeta* 3:e217. 10.1002/imt2.217 39135689 PMC11316920

[B21] Hao Y. Hong Y. Guo H. Qin P. Huang A. Yang X. (2022). Transcriptomic and metabolomic landscape of quinoa during seed germination. *BMC Plant Biol.* 22:237. 10.1186/s12870-022-03621-w 35538406 PMC9088103

[B22] Hassani M. A. Durán P. Hacquard S. (2018). Microbial interactions within the plant holobiont. *Microbiome* 6:58. 10.1186/s40168-018-0445-0 29587885 PMC5870681

[B23] Huang A. C. Jiang T. Liu Y. X. Bai Y. C. Reed J. Qu B. (2019). A specialized metabolic network selectively modulates *Arabidopsis* root microbiota. *Science* 364:eaau6389. 10.1126/science.aau6389 31073042

[B24] Hyatt D. Chen G. L. Locascio P. F. Land M. L. Larimer F. W. Hauser L. J. (2010). Prodigal: Prokaryotic gene recognition and translation initiation site identification. *BMC Bioinform.* 11:119. 10.1186/1471-2105-11-119 20211023 PMC2848648

[B25] Irizarry I. White J. F. (2018). *Bacillus amyloliquefaciens* alters gene expression, ROS production and lignin synthesis in cotton seedling roots. *J. Appl. Microbiol.* 124 1589–1603. 10.1111/jam.13744 29473989

[B26] Khan F. Siddique A. B. Shabala S. Zhou M. Zhao C. (2023). Phosphorus plays key roles in regulating plants’ physiological responses to abiotic stresses. *Plants* 12:2861. 10.3390/plants12152861 37571014 PMC10421280

[B27] Kõljalg U. Larsson K. H. Abarenkov K. Nilsson R. H. Alexander I. J. Eberhardt U. (2005). UNITE: A database providing web-based methods for the molecular identification of ectomycorrhizal fungi. *New Phytol.* 166 1063–1068. 10.1111/j.1469-8137.2005.01376.x 15869663

[B28] Kumar K. Verma A. Pal G. Anubha, White J. F. Verma S. K. (2021). Seed endophytic bacteria of pearl millet (*Pennisetum glaucum* L.) promote seedling development and defend against a fungal phytopathogen. *Front. Microbiol.* 12:774293. 10.3389/fmicb.2021.774293 34956137 PMC8696672

[B29] Kuźniar A. Włodarczyk K. Grza̧dziel J. Woźniak M. Furtak K. Gała̧zka A. (2020). New insight into the composition of wheat seed microbiota. *Int. J. Mol. Sci.* 21:4634. 10.3390/ijms21134634 32629754 PMC7370184

[B30] Lee S. M. Kong H. G. Song G. C. Ryu C. M. (2021). Disruption of firmicutes and *Actinobacteria abundance* in tomato rhizosphere causes the incidence of bacterial wilt disease. *ISME J.* 15 330–347. 10.1038/s41396-020-00785-x 33028974 PMC7852523

[B31] Li D. Liu C. M. Luo R. Sadakane K. Lam T. W. (2015). MEGAHIT: An ultra-fast single-node solution for large and complex metagenomics assembly via succinct de Bruijn graph. *Bioinformatics* 31 1674–1676. 10.1093/bioinformatics/btv033 25609793

[B32] Li L. Yang M. Wei W. Zhao J. Yu X. Impaprasert R. (2023). Characteristics of *Amorphophallus konjac* as indicated by its genome. *Sci. Rep.* 13:22684. 10.1038/s41598-023-49963-9 38114626 PMC10730839

[B33] Li R. Li Y. Kristiansen K. Wang J. (2008). SOAP: Short oligonucleotide alignment program. *Bioinformatics* 24 713–714. 10.1093/bioinformatics/btn025 18227114

[B34] Lindow S. E. Brandl M. T. (2003). Microbiology of the phyllosphere. *Appl. Environ. Microbiol.* 69 1875–1883. 10.1128/AEM.69.4.1875-1883.2003 12676659 PMC154815

[B35] Liu H. Carvalhais L. C. Crawford M. Singh E. Dennis P. G. Pieterse C. M. J. (2017). Inner plant values: Diversity, colonization and benefits from endophytic bacteria. *Front. Microbiol.* 8:2552. 10.3389/fmicb.2017.02552 29312235 PMC5742157

[B36] Magoè T. Salzberg S. L. (2011). FLASH: Fast length adjustment of short reads to improve genome assemblies. *Bioinformatics* 27 2957–2963. 10.1093/bioinformatics/btr507 21903629 PMC3198573

[B37] Malhotra H. Vandana Sharma S. Pandey R. (2018). “Phosphorus nutrition: Plant growth in response to deficiency and excess,” in *Plant nutrients and abiotic stress tolerance*, eds Hasanuzzaman M. Fujita M. Oku H. Nahar K. Hawrylak-Nowak B. (Singapore: Springer), 171–190.

[B38] Martin F. M. Uroz S. Barker D. G. (2017). Ancestral alliances: Plant mutualistic symbioses with fungi and bacteria. *Science* 356:eaad4501. 10.1126/science.aad4501 28546156

[B39] Morella N. M. Weng F. C. Joubert P. M. Metcalf C. J. E. Lindow S. Koskella B. (2020). Successive passaging of a plant-associated microbiome reveals robust habitat and host genotype-dependent selection. *Proc. Natl. Acad. Sci. U. S. A.* 117 1148–1159. 10.1073/pnas.1908600116 31806755 PMC6969547

[B40] Mousa W. K. Shearer C. Limay-Rios V. Ettinger C. L. Eisen J. A. Raizada M. N. (2016). Root-hair endophyte stacking in finger millet creates a physicochemical barrier to trap the fungal pathogen *Fusarium graminearum*. *Nat. Microbiol.* 1:16167. 10.1038/nmicrobiol.2016.167 27669453

[B41] Müller D. B. Vogel C. Bai Y. Vorholt J. A. (2016). The plant microbiota: Systems-level insights and perspectives. *Annu. Rev. Genet.* 50 211–234. 10.1146/annurev-genet-120215-034952 27648643

[B42] Nelson E. B. (2018). The seed microbiome: Origins, interactions, and impacts. *Plant Soil* 422 7–34. 10.1007/s11104-017-3289-7

[B43] Oksanen J. Kindt R. Legendre P. O’Hara R. Stevens M. Oksanen M. (2007). *The vegan package. Community ecology package.* Available online at: http://www.R-project.org (accessed 5 June, 2012).

[B44] Oteino N. Lally R. D. Kiwanuka S. Lloyd A. Ryan D. Germaine K. J. (2015). Plant growth promotion induced by phosphate solubilizing endophytic *Pseudomonas* isolates. *Front. Microbiol.* 6:745. 10.3389/fmicb.2015.00745 26257721 PMC4510416

[B45] Poupin M. J. Ledger T. Roselló-Móra R. González B. (2023). The *Arabidopsis holobiont*: A (re)source of insights to understand the amazing world of plant-microbe interactions. *Environ. Microbiome* 18:9. 10.1186/s40793-023-00466-0 36803555 PMC9938593

[B46] Qi Y. Gao P. Yang S. Li L. Ke Y. Wei H. (2023). Comparative metabolomics analysis reveals dynamic changes in carbohydrate profiles of corms during the “relay growth” of konjac (*Amorphophallus muelleri*). *Front. Plant Sci.* 14:1259561. 10.3389/fpls.2023.1259561 37920719 PMC10619727

[B47] Quast C. Pruesse E. Yilmaz P. Gerken J. Schweer T. Yarza P. (2013). The SILVA ribosomal RNA gene database project: Improved data processing and web-based tools. *Nucleic Acids Res.* 41 D590–D596. 10.1093/nar/gks1219 23193283 PMC3531112

[B48] Ren Y. Yu G. Shi C. Liu L. Guo Q. Han C. (2022). Majorbio cloud: A one-stop, comprehensive bioinformatic platform for multiomics analyses. *Imeta* 1:e12. 10.1002/imt2.12 38868573 PMC10989754

[B49] Roman-Reyn V. Pinilia D. Borjaa F. N. Quiboda I. L. Groen S. C. Mulyaningsihd E. S. (2019). The rice leaf microbiome has a conserved community structure controlled by complex host-microbe. *bioRxiv [Preprint]* 10.1101/615278

[B50] Santolini M. Barabási A. L. (2018). Predicting perturbation patterns from the topology of biological networks. *Proc. Natl. Acad. Sci. U. S. A.* 115 E6375–E6383. 10.1073/pnas.1720589115 29925605 PMC6142275

[B51] Sessitsch A. Pfaffenbichler N. Mitter B. (2019). Microbiome applications from lab to field: Facing complexity. *Trends Plant Sci.* 24 194–198. 10.1016/j.tplants.2018.12.004 30670324

[B52] Shade A. Jacques M. A. Barret M. (2017). Ecological patterns of seed microbiome diversity, transmission, and assembly. *Curr. Opin. Microbiol.* 37 15–22. 10.1016/j.mib.2017.03.010 28437661

[B53] Shahzad R. Khan A. L. Bilal S. Asaf S. Lee I. J. (2018). What is there in seeds? vertically transmitted endophytic resources for sustainable improvement in plant growth. *Front. Plant Sci.* 9:24. 10.3389/fpls.2018.00024 29410675 PMC5787091

[B54] Shakir S. Zaidi S. S. de Vries F. T. Mansoor S. (2021). Plant genetic networks shaping phyllosphere microbial community. *Trends Genet.* 37 306–316. 10.1016/j.tig.2020.09.010 33036802

[B55] Sharma-Poudyal D. Schlatter D. Yin C. Hulbert S. Paulitz T. (2017). Long-term no-till: A major driver of fungal communities in dryland wheat cropping systems. *PLoS One* 12:e0184611. 10.1371/journal.pone.0184611 28898288 PMC5595340

[B56] Simon J. C. Marchesi J. R. Mougel C. Selosse M. A. (2019). Host-microbiota interactions: From holobiont theory to analysis. *Microbiome* 7:5. 10.1186/s40168-019-0619-4 30635058 PMC6330386

[B57] Singh B. K. Trivedi P. (2017). Microbiome and the future for food and nutrient security. *Microb. Biotechnol.* 10 50–53. 10.1111/1751-7915.12592 28074557 PMC5270726

[B58] Singh B. K. Trivedi P. Egidi E. Macdonald C. A. Delgado-Baquerizo M. (2020). Crop microbiome and sustainable agriculture. *Nat. Rev. Microbiol.* 18 601–602. 10.1038/s41579-020-00446-y 33037425

[B59] Toju H. Peay K. G. Yamamichi M. Narisawa K. Hiruma K. Naito K. (2018). Core microbiomes for sustainable agroecosystems. *Nat. Plants* 4 247–257. 10.1038/s41477-018-0139-4 29725101

[B60] Trivedi P. Leach J. E. Tringe S. G. Sa T. Singh B. K. (2020). Plant-microbiome interactions: From community assembly to plant health. *Nat. Rev. Microbiol.* 18 607–621. 10.1038/s41579-020-0412-1 32788714

[B61] Truyens S. Weyens N. Cuypers A. Vangronsveld J. (2015). Bacterial seed endophytes: Genera, vertical transmission and interaction with plants. *Environ. Microbiol. Rep.* 7 40–50. 10.1111/1758-2229.12181

[B62] Turner T. R. James E. K. Poole P. S. (2013). The plant microbiome. *Genome Biol.* 14:209. 10.1186/gb-2013-14-6-209 23805896 PMC3706808

[B63] Vacher C. Hampe A. Porté A. J. Sauer U. Compant S. Morris C. E. (2016). The phyllosphere: Microbial jungle at the plant–climate interface. *Annu. Rev. Ecol. Evol. Systemat.* 47 1–24. 10.1146/annurev-ecolsys-121415-032238

[B64] Vandenkoornhuyse P. Quaiser A. Duhamel M. Le Van A. Dufresne A. (2015). The importance of the microbiome of the plant holobiont. *New Phytol.* 206 1196–1206. 10.1111/nph.13312 25655016

[B65] Verzeaux J. Alahmad A. Habbib H. (2016). Cover crops prevent the deleterious effect of nitrogen fertilization on bacterial diversity by maintaining the carbon content of plowed soil. *Geoderma* 281 49–57. 10.1016/j.geoderma.2016.06.035

[B66] Vorholt J. A. Vogel C. Carlström C. I. Müller D. B. (2017). Establishing causality: Opportunities of synthetic communities for plant microbiome research. *Cell Host Microbe* 22 142–155. 10.1016/j.chom.2017.07.004 28799900

[B67] Wagg C. Schlaeppi K. Banerjee S. Kuramae E. E. van der Heijden M. G. A. (2019). Fungal-bacterial diversity and microbiome complexity predict ecosystem functioning. *Nat. Commun.* 10:4841. 10.1038/s41467-019-12798-y 31649246 PMC6813331

[B68] Wang C. N. Qin Y. F. Li Y. L. Wu R. L. Zhu D. Q. Zhou F. (2021). Variations of root-associated bacterial cooccurrence relationships in paddy soils under chlorantraniliprole (CAP) stress. *Sci. Total Environ.* 779:146247. 10.1016/j.scitotenv.2021.146247 33743468

[B69] Wang F. Men X. Zhang G. Liang K. Xin Y. Wang J. (2018). Assessment of 16S rRNA gene primers for studying bacterial community structure and function of aging flue-cured tobaccos. *AMB Exp.* 8:182. 10.1186/s13568-018-0713-1 30415449 PMC6230335

[B70] Wang H. Narsing Rao M. P. Gao Y. Li X. Gao R. Xie Y. (2021). Insights into the endophytic bacterial community comparison and their potential role in the dimorphic seeds of halophyte Suaeda glauca. *BMC Microbiol.* 21:143. 10.1186/s12866-021-02206-1 33980153 PMC8114534

[B71] Wei H. Wang L. Hassan M. Xie B. (2018). Succession of the functional microbial communities and the metabolic functions in maize straw composting process. *Bioresour. Technol.* 256 333–341. 10.1016/j.biortech.2018.02.050 29459320

[B72] Wei H. Yang M. Ke Y. Liu J. Chen Z. Zhao J. (2022). Comparative physiological and transcriptomic profiles reveal regulatory mechanisms of soft rot disease resistance in *Amorphophallus spp*. *Physiol. Mol. Plant Pathol.* 118:101807. 10.1016/j.pmpp.2022.101807

[B73] Weitbrecht K. Müller K. Leubner-Metzger G. (2011). First off the mark: Early seed germination. *J. Exp. Bot.* 62 3289–3309. 10.1093/jxb/err030 21430292

[B74] Wen Y. Wei T. Z. Luo Z. J. Hu W. X. Chen Y. Dai Q. L. (2023). Effects of two salt tolerant *Pseudomonas* strains on the germination of rapeseed seeds under salt stress. *Bot. Res.* 12 192–199. 10.12677/BR.2023.124026

[B75] Weyens N. van der Lelie D. Taghavi S. Newman L. Vangronsveld J. (2009). Exploiting plant-microbe partnerships to improve biomass production and remediation. *Trends Biotechnol.* 27 591–598. 10.1016/j.tibtech.2009.07.006 19683353

[B76] Xiong C. Singh B. K. He J. Z. Han Y. L. Li P. P. Wan L. H. (2021a). Plant developmental stage drives the differentiation in ecological role of the maize microbiome. *Microbiome* 9:171. 10.1186/s40168-021-01118-6 34389047 PMC8364065

[B77] Xiong C. Zhu Y. G. Wang J. T. Singh B. Han L. L. Shen J. P. (2021b). Host selection shapes crop microbiome assembly and network complexity. *New Phytol.* 229 1091–1104. 10.1111/nph.16890 32852792

[B78] Yang M. Gao P. Guo J. Qi Y. Li L. Yang S. (2024). The endophytic fungal community plays a crucial role in the resistance of host plants to necrotic bacterial pathogens. *Physiol. Plant* 176:e14284. 10.1111/ppl.14284 38618747

[B79] Yang M. Qi Y. Liu J. Gao P. Huang F. Yu L. (2023). Different response mechanisms of rhizosphere microbial communities in two species of *Amorphophallus* to *Pectobacterium carotovorum* subsp. carotovorum infection. *Plant Pathol J.* 39 207–219. 10.5423/PPJ.OA.12.2022.0157 37019830 PMC10102568

[B80] Yang M. Qi Y. Liu J. Wu Z. Gao P. Chen Z. (2022). Dynamic changes in the endophytic bacterial community during maturation of *Amorphophallus muelleri* seeds. *Front. Microbiol.* 13:996854. 10.3389/fmicb.2022.996854 36225382 PMC9549114

[B81] Zayed O. Hewedy O. A. Abdelmoteleb A. Ali M. Youssef M. S. Roumia A. F. (2023). Nitrogen journey in plants: From uptake to metabolism, stress response, and microbe interaction. *Biomolecules* 13:1443. 10.3390/biom13101443 37892125 PMC10605003

[B82] Zhang C. M. Xu M. J. Li X. W. Xing K. Qin S. (2022). Recent research advances and application potential in agriculture of *Pseudomonas chlororaphis*. *Acta Microbiol. Sinica* 62 391–402. 10.13343/j.cnki.wsxb.20210198

[B83] Zhang X. Ma Y. N. Wang X. Liao K. He S. Zhao X. (2022). Dynamics of rice microbiomes reveal core vertically transmitted seed endophytes. *Microbiome* 10:216. 10.1186/s40168-022-01422-9 36482381 PMC9733015

[B84] Zhao Y. Yang M. Qi Y. Gao P. Ke Y. Liu J. (2024). Combined analysis of the metabolome and transcriptome sheds new light on the mechanisms of seed maturation in *Amorphophallus muelleri*. *J. Plant Growth Regul.* 43 4263–4278. 10.1007/s00344-024-11390-z

