# Supplementary material for: Plant developmental stage drives the assembly and functional adaptability of endophytic microbial communities
Source: Front Microbiol. 2025 May 29;16:1492141. doi: 10.3389/fmicb.2025.1492141 (PMC12158941; doi:10.3389/fmicb.2025.1492141)
Supplement: Supplementary file 1 [file Data_Sheet_1.docx]

**<Supplementary figures>**


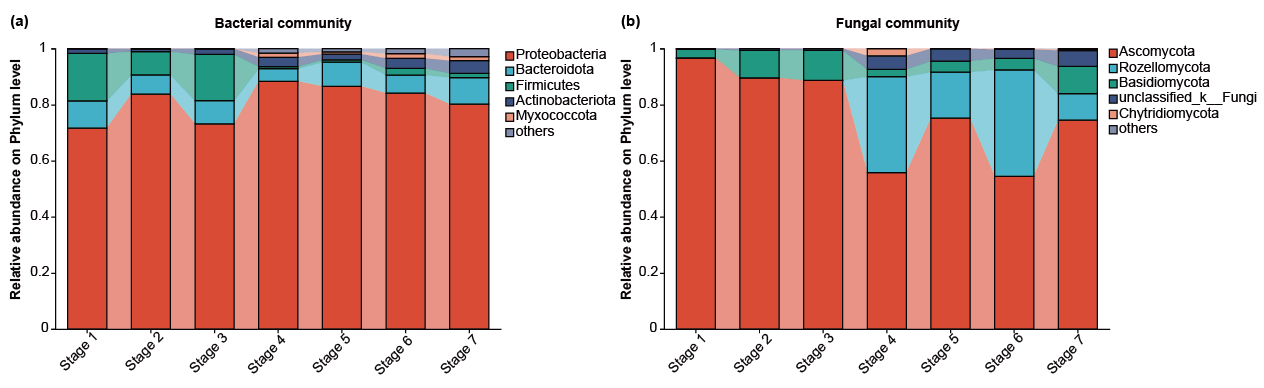


**Figure S1** Composition and relative abundance of major bacterial (**a**) and fungal (**b**) taxa in different developmental stages of seeds and seedlings at the phylum level. Phyla less than 1% of the total reads were grouped into “others”.


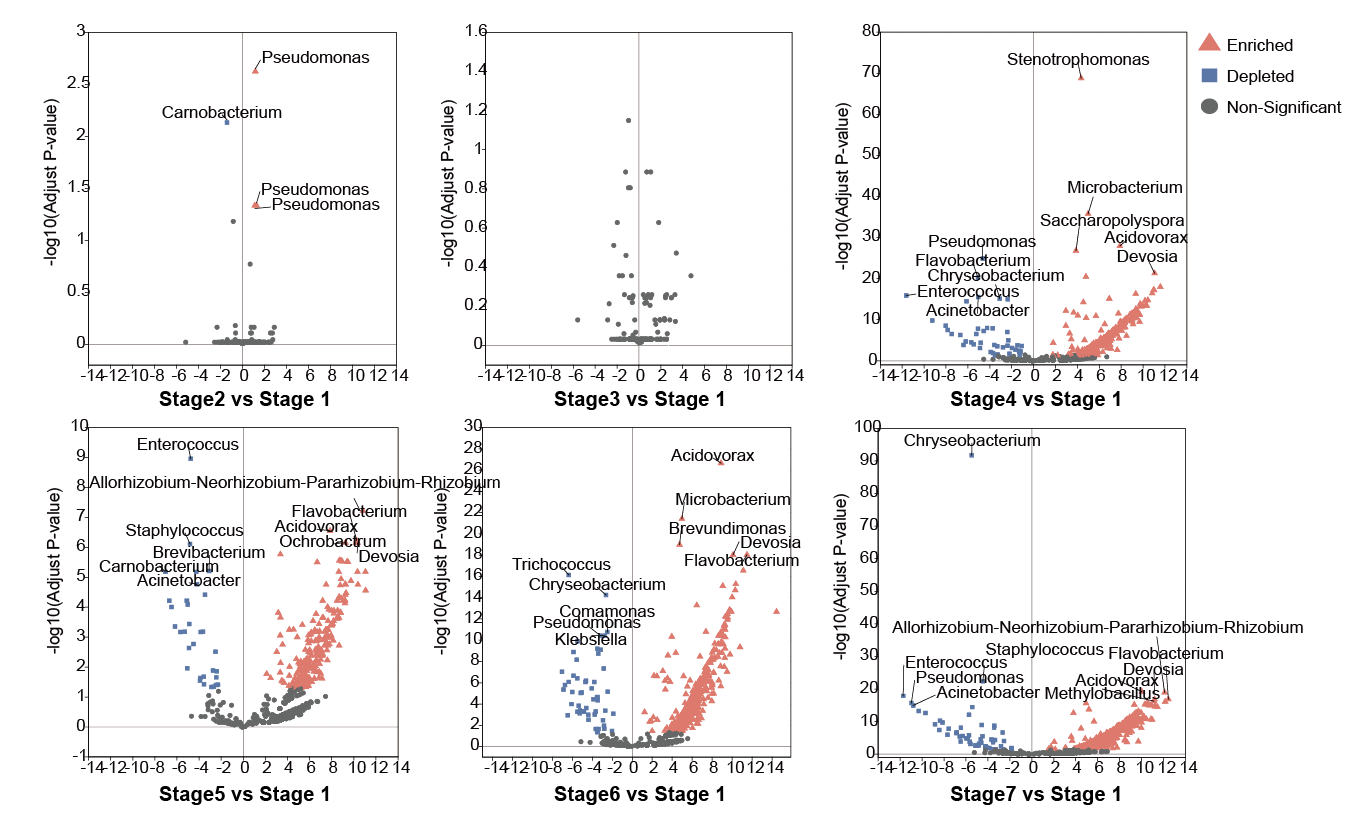
**Figure S2** Volcano plot illustrating the enrichment and depletion patterns of bacterial microbiomes in the different developmental stages compared with the Stage 1. Triangles represent significantly enriched OTUs, while squares represent significantly depleted OTUs. Only the top 5 OTUs in terms of enrichment/depletion are labeled with their taxonomic names in the figure.

**
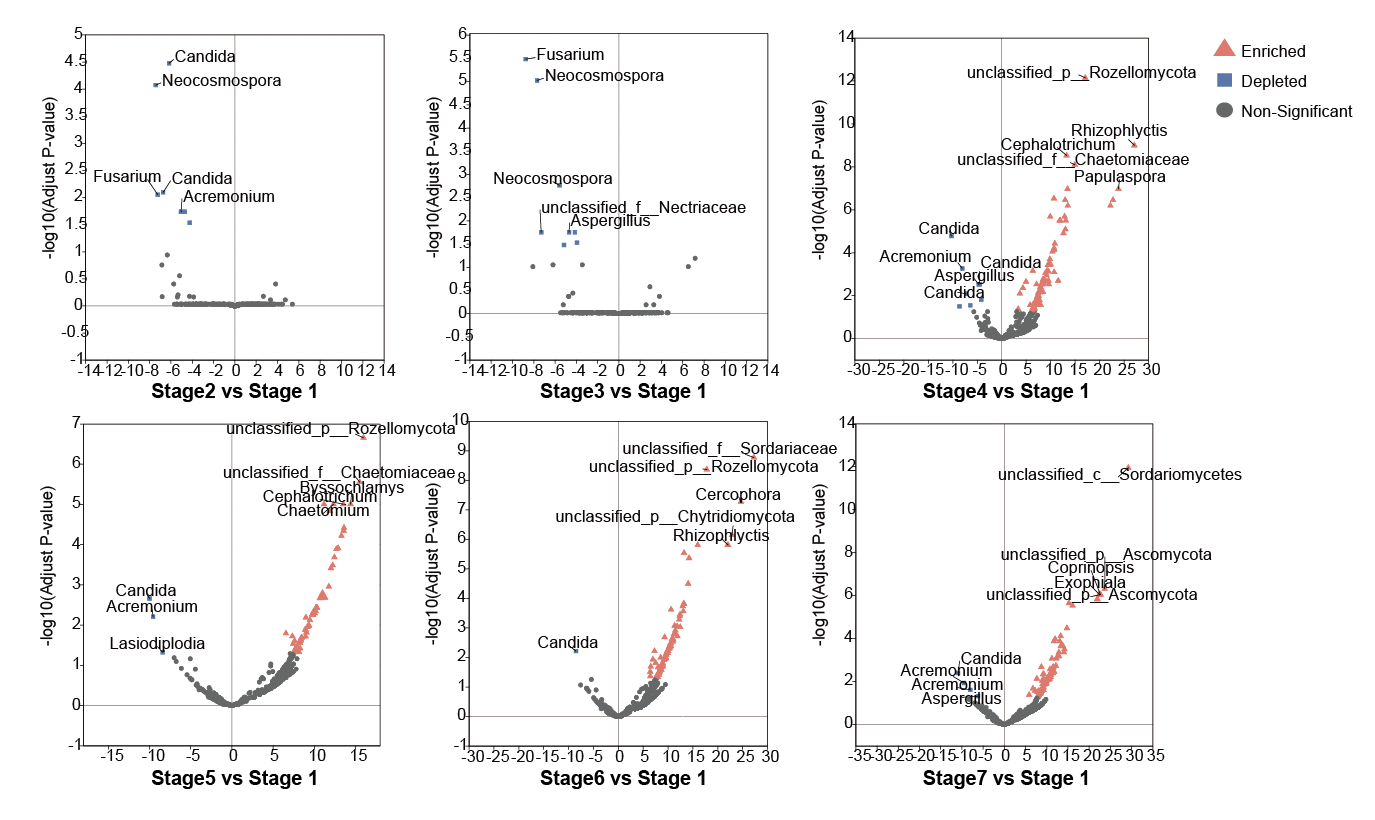
Figure S3** Volcano plot illustrating the enrichment and depletion patterns of fungal microbiomes in the different developmental stages compared with the Stage 1. Triangles represent significantly enriched OTUs, while squares represent significantly depleted OTUs. Only the top 5 OTUs in terms of enrichment/depletion are labeled with their taxonomic names in the figure.

**<Supplementary tables>**

**Table S1** The PCR amplification conditions in this study.

| **Gene fragment** | **Primers** | **Sequence (5’-3’)** | **PCR program** |
| --- | --- | --- | --- |
| ITS | ITS1F | CTTGGTCATTTAGAGGAAGTAA | pre-denaturation at 95℃ for 3 min, 35 cycles of 95℃ for 30 s, 55℃ for 30 s and 72℃ for 45 s, a final elongation at 72℃ for 10 min. |
|  | ITS2R | GCTGCGTTCTTCATCGATGC |  |
| 16S rRNA | 799F | AACMGGATTAGATACCCKG | pre-denaturation at 95°C for 3 min, 27 cycles of 95°C for 30 s, 55°C for 30 s, and 72°C for 45 s, a final elongation at 72°C for 10 min. |
|  | 1392R | ACGGGCGGTGTGTRC |  |
|  | 799F | AACMGGATTAGATACCCKG | pre-denaturation at 95°C for 3 min, 13 cycles of 95°C for 30 s, 55°C for 30 s, and 72°C for 45 s, a final elongation at 72°C for 10 min. |
|  | 1193R | ACGTCATCCCCACCTTCC |  |

**Table S2** The relative abundances of endophytic bacterial communities on phylum level at different treatments (±SEM, n = 3/treatment, %)

| **Treatment** | **Proteobacteria** | **Bacteroidota** | **Firmicutes** | **Actinobacteriota** | **Myxococcota** | **Acidobacteriota** | **others** |
| --- | --- | --- | --- | --- | --- | --- | --- |
| Stage 1 | 71.64±3.31c | 9.70±2.60 a | 17.01±2.73 a | 1.53±0.23 c | 0 c | 0 b | 0.13±0.04 c |
| Stage 2 | 83.79±2.39 ab | 6.84±0.39 a | 8.29±2.63 b | 1.01±0.18 c | 0 c | 0 b | 0.07±0.03 c |
| Stage 3 | 73.14±1.52 c | 8.29±2.14 a | 16.48±3.16 a | 1.97±0.49 bc | 0 c | 0 b | 0.11±0.01 c |
| Stage 4 | 88.34±3.20 a | 4.48±0.87 a | 0.74±0.19 c | 3.35±1.09 ab | 1.50±0.64 a | 0.06±0.01 b | 1.53±0.45 ab |
| Stage 5 | 86.54±5.43 a | 8.60±6.96 a | 0.82±0.33 c | 2.06±1.49 bc | 0.80±0.56 b | 0.04±0.02 b | 1.14±0.89 b |
| Stage 6 | 84.09±1.93 ab | 6.49±0.66 a | 2.39±1.08 c | 3.52±0.85 ab | 1.68±0.02 a | 0.10±0.02 b | 1.74±0.01 ab |
| Stage 7 | 80.16±1.96 b | 9.50±2.95 a | 1.50±0.46 c | 4.52±0.93 a | 1.47±0.28 a | 0.69±0.81 a | 2.15±0.28 a |

Significant differences among treatments are shown by different lowercase letters within the column, according to a least significant difference test (LSD; p < 0.05).

**Table S3** The relative abundances of endophytic fungal communities on phylum level at different treatments (±SEM, n = 3/treatment, %)

| **Treatment** | **Ascomycota** | **Rozellomycota** | **Basidiomycota** | **unclassified_k__Fungi** | **Chytridiomycota** | **others** |
| --- | --- | --- | --- | --- | --- | --- |
| Stage 1 | 96.64±1.12 a | 0.03±0.003 c | 3.07±0.98 b | 0.27±0.13 b | 0 a | 0.004±0.006 b |
| Stage 2 | 89.58±0.54 a | 0 c | 9.78±0.44 a | 0.63±0.12 b | 0 a | 0 b |
| Stage 3 | 88.64±1.19 a | 0 c | 10.76±1.29 a | 0.59±0.10 b | 0 a | 0 b |
| Stage 4 | 55.62±11.86 c | 34.43±16.96 a | 2.55±1.07 b | 4.80±2.87 a | 2.57±4.26 a | 0.019±0.003 b |
| Stage 5 | 75.28±6.03 b | 16.36±4.32 b | 3.95±1.50 b | 4.33±2.05 a | 0.05±0.027 a | 0.025±0.029 b |
| Stage 6 | 54.52±7.78 c | 37.86±10.53 a | 4.12±2.22 b | 3.19±0.71 ab | 0.29±0.20 a | 0.013±0.013 b |
| Stage 7 | 74.48±5.23 b | 9.44±2.17 b | 9.68±3.09 a | 5.70±2.22 a | 0.36±0.28 a | 0.336±0.289 a |

Significant differences among treatments are shown by different lowercase letters within the column, according to a least significant difference test (LSD; p < 0.05).

**Table S4** Topology properties of the co-occurrence network of microbial communities in the seeds or seedlings of *A. muelleri* at different developmental stages.

| **Networks** | **Group** | **node** | **edge** | **positive** | **negative** | **Average Degree** |
| --- | --- | --- | --- | --- | --- | --- |
| bacterial-bacterial | Stage 1 | 50 | 251 | 67.33% | 32.67% | 10.040 |
|  | Stage 2 | 44 | 164 | 78.05% | 21.95% | 7.455 |
|  | Stage 3 | 45 | 211 | 66.82% | 33.18% | 9.378 |
|  | Stage 4 | 219 | 2429 | 72.38% | 27.62% | 22.183 |
|  | Stage 5 | 228 | 5655 | 59.61% | 40.39% | 49.605 |
|  | Stage 6 | 276 | 3785 | 51.52% | 48.48% | 27.428 |
|  | Stage 7 | 363 | 8330 | 51.98% | 48.02% | 45.895 |
| fungal–fungal | Stage 1 | 93 | 1370 | 66.06% | 33.94% | 29.462 |
|  | Stage 2 | 118 | 1729 | 58.76% | 41.24% | 29.305 |
|  | Stage 3 | 108 | 1204 | 60.80% | 39.20% | 22.296 |
|  | Stage 4 | 202 | 4750 | 72.78% | 27.22% | 47.030 |
|  | Stage 5 | 198 | 5285 | 70.43% | 29.57% | 53.384 |
|  | Stage 6 | 187 | 3819 | 51.77% | 48.23% | 40.845 |
|  | Stage 7 | 236 | 8909 | 56.35% | 43.65% | 75.500 |
| bacterial–fungal | Stage 1 | 145 | 1061 | 37.42% | 62.58% | 14.634 |
|  | Stage 2 | 164 | 1132 | 40.02% | 29.98% | 13.805 |
|  | Stage 3 | 155 | 984 | 56.61% | 43.39% | 12.697 |
|  | Stage 4 | 431 | 7255 | 31.73% | 68.27% | 33.666 |
|  | Stage 5 | 429 | 9516 | 35.69% | 64.31% | 44.364 |
|  | Stage 6 | 470 | 7725 | 51.31% | 48.69% | 32.872 |
|  | Stage 7 | 599 | 18239 | 48.62% | 51.38% | 60.898 |

**Table S5** Sequencing statistics of the metagenomic libraries.

| Samples | Raw reads | Clean reads | Optimized reads | Contigs | ORFs |
| --- | --- | --- | --- | --- | --- |
| Stage 1_1 | 86166422 | 85149920 | 14506088 | 148857 | 234127 |
| Stage 1_2 | 87627044 | 86476966 | 8070192 | 110379 | 163465 |
| Stage 1_3 | 82681446 | 81534444 | 16304490 | 159160 | 244481 |
| Stage 2_1 | 88705274 | 87478748 | 11172492 | 143063 | 212430 |
| Stage 2_2 | 83907570 | 82883514 | 9568156 | 116177 | 174224 |
| Stage 2_3 | 89776582 | 88224690 | 11812524 | 139034 | 207105 |
| Stage 3_1 | 84776602 | 83590646 | 5384792 | 78314 | 111890 |
| Stage 3_2 | 94354616 | 92394388 | 2867208 | 46425 | 62513 |
| Stage 3_3 | 88128406 | 87097648 | 5778998 | 78221 | 114459 |
| Stage 4_1 | 87105462 | 85535894 | 84399992 | 940429 | 1187625 |
| Stage 4_2 | 94819652 | 93273206 | 91173034 | 1057975 | 1339082 |
| Stage 4_3 | 91246732 | 89449360 | 86918462 | 1008778 | 1279409 |
| Stage 5_1 | 87417106 | 85649802 | 82477600 | 931536 | 1170056 |
| Stage 5_2 | 84775438 | 82957892 | 79982144 | 916467 | 1159203 |
| Stage 5_3 | 91222800 | 89529950 | 86285584 | 1000492 | 1257428 |
| Stage 6_1 | 90663430 | 88541794 | 84125950 | 958648 | 1210380 |
| Stage 6_2 | 98898426 | 97167044 | 94858924 | 1083957 | 1368153 |
| Stage 6_3 | 92769710 | 90940938 | 88752664 | 1020233 | 1288304 |
| Stage 7_1 | 92746822 | 90854950 | 86816544 | 910857 | 1146853 |
| Stage 7_2 | 97056542 | 95089652 | 91052690 | 1013685 | 1266716 |
| Stage 7_3 | 85681702 | 83958236 | 80632468 | 878651 | 1099678 |
